# Successful treatment of acute promyelocytic leukemia in a patient undergoing hemodialysis with arsenic trioxide

**DOI:** 10.1002/ccr3.4300

**Published:** 2021-07-23

**Authors:** Akiko Hashimoto, Yasuhiro Tanaka, Isaku Shinzato

**Affiliations:** ^1^ Department of Hematology and Clinical Immunology Kobe City Nishi‐Kobe Medical Center Kobe Japan

**Keywords:** acute promyelocytic leukemia, arsenic trioxide, chronic kidney disease

## Abstract

A man undergoing hemodialysis was diagnosed with acute promyelocytic leukemia (APL). He received arsenic trioxide as a single agent and achieved complete molecular remission without severe adverse events. Arsenic trioxide can be used safely and effectively for patients with APL under hemodialysis.

## Graphical abstract

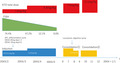

## 1 INTRODUCTION

Acute promyelocytic leukemia (APL) is a rare subtype of acute myeloid leukemia defined by the fusion gene of the promyelocytic leukemia (*PML*) gene with the retinoic acid receptor α (*RARA*) gene as a result of a balanced translocation between chromosome 15 and 17. Previously, the prognosis of APL was poor because of bleeding tendency due to disseminated intravascular coagulation, but the prognosis of APL was dramatically improved by following the introduction of all‐trans retinoic acid (ATRA) to the induction chemotherapy for APL. According to NCCN guidelines, the standard treatment of low‐risk APL is currently a combination therapy of ATRA and arsenic trioxide (ATO). If ATO is not available, ATRA combination treatment with anthracycline or gemtuzumab ozogamicin (GO) is useful.

Currently, no standard strategy for APL complicated with organ failure is established. In cases complicated with chronic kidney disease (CKD), ATRA is prohibited for patients with APL undergoing hemodialysis in Japan owing to the risk of hypervitaminosis A. Here, we report the case of APL in a patient with CKD treated with ATO as a single agent. He achieved molecular complete remission (CR) with ATO. Thus, we consider that this is one of the new treatment strategies for APL in patients undergoing hemodialysis.

## 2 CASE PRESENTATION

**FIGURE 1:**
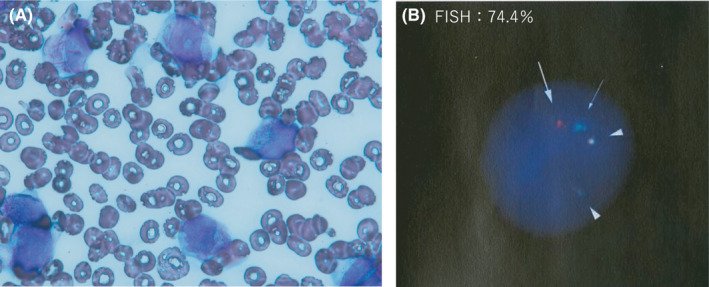
Acute promyelocytic leukemia at diagnosis. A, Bone marrow smear, (B) FISH (Fluorescence in situ hybridization) analysis of metaphase spreads and interphase nuclei of bone marrow cells, a signal of *PML* (15q22) probe (arrow), a signal of *RARA* (17q21) probe (thin arrow), two signals of *PML/RARA* probe (arrowhead) G‐banded karyotype of bone marrow cells: 46, XY, t(15;17)(q22;q21)[8]/46,XY[12]

A 53‐year‐old man was admitted to a different hospital because of pancytopenia. He has been on continuous hemodialysis since 5 years ago for chronic kidney disease (CKD) that had developed because of polycystic kidney disease. Pancytopenia was detected on routine examination during hemodialysis. On admission, he was asymptomatic. His laboratory data showed the following white blood cell count; 1000/μL, including 0.5% promyelocytes with the Auer body, red blood cell count 3.27 × 10^6^/μL, hemoglobin level 10.5 g/dL, platelet count 12.6 × 10^4^/μL, C‐reactive protein level 0.09 mg/dL, lactate dehydrogenase level 147 IU/L, blood urea nitrogen level 32 mg/dL, and creatinine level 9.64 mg/dL. Bone marrow examination showed that many abnormal promyelocytes were found in the smear specimen (Figure 1A). Fluorescence in situ hybridization (FISH) analysis showed 74.4% fusion signals between the *PML* and *RARA* probes (Figure 1B). Reverse transcriptase‐polymerase chain reaction (RT‐PCR) confirmed the presence of *PML*‐*RARA* fusion transcript. Chromosomal analysis using G‐banding showed 46,XY,t(15;17)(q22;q21)[8]/46,XY[12]. Thus, he was diagnosed with acute promyelocytic leukemia (APL). He was categorized as at a low risk in accordance with the PETHEMA (Programa para el Estudio de la Terapeutica en Hemopatia maligna) criteria. ATRA was prohibited for hemodialysis‐dependent CKD patients in Japan; therefore, he was given induction therapy that consisted of only intravenous arsenic trioxide (ATO) at 0.1 mg/kg body weight after hemodialysis every other day. He was on dialysis for three hours a day. He had end‐stage renal disease and had been undergoing intermittent infusion hemodiafiltration since 2015. The vascular access was through the venous‐arterial fistula on the left arm, and the dialyzer was used as Toraylight HDF^®^, with a membrane area of 2.1 m^2^, blood flow of 250 mL per minutes, and dialysate flow of 500 mL per minute. The anticoagulant agent was heparin of 1250 unit bolus and administered at 750 units per hour.

**FIGURE 2:**
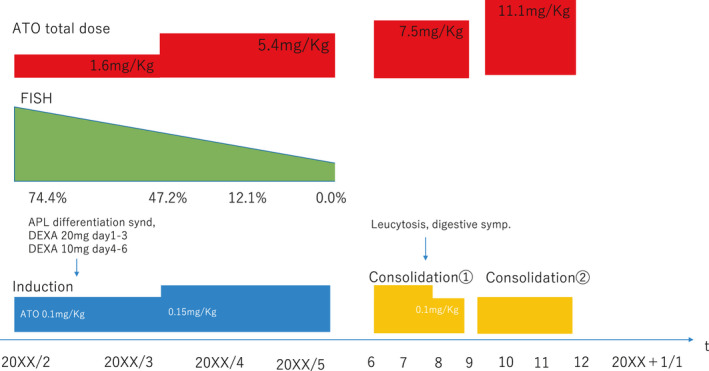
Clinical course

Serum potassium and magnesium levels were maintained at >4.0 mmol/L and 2.0 mEq/L, respectively. The QTc duration was monitored by electrocardiography twice a week. Two weeks after the induction therapy, he developed symptoms of APL differentiation syndrome, such as fever, mild hypoxia, and hyperleukocytosis. His body temperature was reduced and hypoxia was improved by the administration of dexamethasone at 20 mg/d for 3 days and 10 mg/d for the next 3 days. ATO was administered for 4 weeks (total dose of 110 mg/Kg body weight), and he achieved hematological complete remission. However, FISH analysis detected 47.2% of fusion signals between the *PML* and *RARA* probes in his bone marrow, suggesting that he did not achieve cytogenetic remission. Then, we decided to increase the dose of ATO to 0.15 mg/kg every other day. After three months of induction therapy (total dose of 357 mg/body), bone marrow examination showed 0.3% of promyelocytes in his smear specimen; chromosomal analysis using G‐banding showed 46, XY[20/20]; no fusion signal between the *PML* and *RARA* probes was detected by FISH analysis; and no fusion transcript was detected by RT‐PCR. These results suggested that he achieved molecular complete remission. During ATO administration, his electrolytes and QTc duration were kept stable by electrocardiography; however, he complained of intermittent gastrointestinal symptoms, such as abdominal distension and pain. We considered these symptoms to be the adverse effects of ATO; thus, we reduced the ATO dose from 0.15 mg/kg to 0.1 mg/kg during the first course of consolidation therapy (total dose of 495 mg/body). After the dose reduction of ATO, his gastrointestinal symptoms resolved, and the effect of ATO was maintained. Therefore, we continued to administer 0.1 mg/kg ATO. After the first course of consolidation therapy, he received the second course of consolidation therapy after an interval of 1 month and the third consolidation after an interval of 1 year (Figure 2). At the time of writing this manuscript, he had maintained complete molecular remission for more than 2 years.

## 3 DISCUSSION

To the best of our knowledge, this is the first case wherein ATO was used as a single agent for a patient with APL undergoing hemodialysis to achieve and maintain molecular CR. Patients with APL may experience a life‐threatening complication early at diagnosis; however, patients who survive generally have a very favorable prognosis after treatment with ATO and ATRA.^1^, ^2^ According to the NCCN guidelines,^3^ the current standard treatment for APL is not only ATRA and ATO combination therapy, but also ATRA and anthracycline combination therapy, such as idarubicin or ATRA and gemtuzumab ozogamicin combination therapy. Currently, there are no standard treatment guidelines for APL in patients undergoing hemodialysis. Although the combination therapy with ATRA is a standard treatment for APL, it is prohibited in Japan because of the risk of hypervitaminosis A in patients with APL undergoing hemodialysis. In the case of patients with CKD, ATRA may be overdosed because ATRA is known to be metabolized and excreted in a glucuronide form in the bile and urine^4^, ^5^ and ATRA is not removed during hemodialysis. Hypervitaminosis A may be fatal. Some studies have shown CR with the use of ATRA for patients with APL undergoing hemodialysis.^6^, ^7^, ^8^ However, ATRA is not removed by hemodialysis; therefore, the pharmacokinetics of ATRA are not stable in patients with APL undergoing hemodialysis,^9^ and the efficacy of ATRA is uncertain. ATO can be removed by hemodialysis,^9^ and the serum ATO concentration normalizes and stabilizes after 6 months.^10^ One of the standard treatments for APL is a combination of ATRA and anthracycline. Anthracycline anticancer drugs are excreted in the liver, and this is a useful option for APL patients with renal impairment. However, ATRA is prohibited in Japan, and it has been often shown that myelosuppression and febrile neutropenia are the major adverse effects of anthracycline. The long‐term prognosis of APL is favorable in the combination therapy of ATRA and anthracycline, but it is unclear that the long‐term prognosis of APL treated with anthracycline as a single agent. Moreover, there has been no report on whether the combination therapy of ATO and anthracycline is effective for APL patients. Thus, we decided to use ATO as a single agent. ATO can be used repeatedly for patients with APL undergoing hemodialysis; therefore, we consider that ATO is suitable for patients with APL undergoing hemodialysis. I have summarized ATO treatment for APL in Table 1.

**TABLE 1:** Comparison of treatments for acute promyelocytic leukemia

Reference	Year	Induction therapy	Consolidation therapy and maintenance therapy	Outcome
NCCN guideline^3^	2021	0.15 mg/Kg/d of Arsenic trioxide	Arsenic trioxide 0.15 mg/Kg 5/wk for 4 wk every 8 wk for total four cycles	–
		+45 mg/m^2^/d of ATRA until CR or until 60 times of arsenic trioxide administration	+ATRA 45 mg/m^2^/d for 2 wk every 4 wk for seven cycles	
Mozafar Aznab^20^	2017	0.15 mg/Kg/d of ATO until CR	0.15 mg/Kg/d of ATO for 28 d as a consolidation therapy	Mean DFS 101 mo and 97 mo (male and female)
			The same dose of ATO was given every 3‐4 mo for 14 d for 2 y as a maintenance therapy	
Yamamoto et al^14^	2009	ATRA 70 mg/body + 240 mg/m^2^ of behenoyle AraC for 5 d and 30 mg/m^2^ of Daunorubicin	0.15 mg/Kg/d of ATO	
Vikram et al^11^	2006	0.15 mg/Kg/d of ATO until CR	0.15 mg/Kg/d of ATO for 4 wk as a consolidation therapy	3‐y EFS 74.87% ± 5.6%
			0.15 mg/Kg/d of ATO for 10 d a month for 6 mo as a maintenance therapy	3‐y DFS
				87.21%±4.93%
				3‐year OS
				86.11% ± 4.08%

Abbreviations: ATO, arsenic trioxide; ATRA, all‐trans retinoic acid.

Some studies have shown that ATO as a single agent can achieve a high CR rate in APL. Vikram Mathew et al used ATO in 72 newly diagnosed patients (patients without organ failure) and reported complete hematological remission in 86.1% of the patients.^11^ They also reported that the three‐year Kaplan‐Meier estimates of event‐free survival (EFS), disease‐free survival (DFS), and overall survival (OS) were 74.8%, 87.21%, and 86.11%, respectively, at a median follow‐up of 25 months. Shen et al^12^ described the outcomes of ATO‐based treatment in 15 relapsed APL patients: 10 patients achieved CR with the use of ATO as a single agent. In particular, low‐ and intermediate‐risk groups, according to the PETHEMA criteria, tend to achieve more benefits than the high‐risk groups.^13^ Our patient was categorized as being at low risk according to the PETHEMA criteria; therefore, we decided to treat our patient with ATO as a single agent. Major adverse events of ATO include APL differentiation syndrome, electrode abnormality, and long QT syndrome. Yamamoto et al^14^ reported that ATO can be safely used for some APL patients undergoing hemodialysis by monitoring plasma arsenic concentrations; they measured the plasma ATO concentration and managed the adverse events. However, we cannot measure the plasma ATO concentration at our hospital. Therefore, we regularly check the electrolytes and electrocardiograms and manage these advert events as long as the toxicity of ATO is mild and reversible.

There is one issue that needs to be considered while administering ATO for APL. There are limited reports on the long‐term outcome of using single‐agent ATO in the management of APL. Furthermore, it is unclear how many courses of consolidation therapy with ATO are required to treat patients with APL undergoing hemodialysis. Lo‐Coco et al reported that ATRA/ATO combination therapy may be superior to ATRA + IDR as the induction therapy and four sessions of consolidation therapy.^15^ The NCCN guidelines recommend four sessions of consolidation therapy.^16^ However, there is no consensus regarding the optimal number of consolidation therapy sessions for the treatment of APL with ATO as a single agent. In particular, the case of our patient was complicated with CKD. G.S Emmons et al reported that a patient with APL undergoing hemodialysis achieved CR with ATO as a single agent and maintained CR for three years with combination therapy of ATO and idarubicin.^17^ Although the conventional dose of ATO is 0.15 mg/Kg, they used 0.1 mg/Kg of ATO. They mentioned in Discussion that as the ATO concentration was not assessed or monitored, the titration of ATO was based on the toxicity profile. The suitable dosage of ATO for patients with CKD is unclear. We also started at an initial dose of 0.1 mg/Kg and increased the dose to 0.15 mg/Kg, which still showed an insufficient effect. One of the standard treatments for APL is a combination of ATRA and anthracycline. Anthracycline anticancer drugs are excreted in the liver, and this is a useful option for APL patients with renal impairment. However, ATRA is prohibited in Japan, and it has been often shown that myelosuppression and febrile neutropenia are a major advert effect in anthracycline. And the long‐term prognosis of APL is favorable in the combination therapy of ATRA and anthracycline, but it is unclear about the long‐term prognosis of APL in the treatment of anthracycline as a single agent. Also there has not been reported that the combination therapy of ATO and anthracycline is effective for APL patients. Thus, we decided to use ATO as a single agent. Since the usefulness of ATO was shown even in patients with relapsed APL,^18^, ^19^ we decided to continue to use ATO by increasing its dosage. We decided to start at a low dose of 0.1mg/Kg and increased to 0.15 mg/Kg if no adverse events occurred. This patient was treated with ATO as a single agent for the induction therapy; however, consolidation therapy involved not only ATO, but also idarubicin. Previously, two studies have shown the long‐term outcome of using a single agent, ATO, in APL treatment.^11^, ^20^ Vikram Mathews et al reported the efficacy and minimal toxicity of ATO in a newly diagnosed APL patient with the following regimen. ATO was administered at 10 mg daily dose for adults and 0.15 mg/kg for pediatric patients until CR was achieved. Another 4‐week course was administered after a 4‐week interval as consolidation therapy to those who had achieved CR. Subsequently, after a second 4‐week interval, it was administered for 10 days/month for 6 months as maintenance therapy. Mozaffar Aznab and Mabdour Rezzael reported the results of induction, consolidation, and maintenance therapies with ATO as a single agent in an 11‐year follow‐up. They reported that ATO was infused at a daily dose of 0.15 mg/kg as induction therapy until CR was achieved. After 2 weeks of rest, ATO was administered daily for 28 days as consolidation therapy. Then, ATO was administered for 14 days every 3‐4 months for 2 years. Both reports differed in the method of consolidation and maintenance therapies; however, they reported a CR rate and long‐term survival rate of >80%. Further research on a larger sample is necessary to establish the methods of consolidation and maintenance therapies with ATO as a single agent.

In summary, single‐agent ATO is useful for treating patients with APL undergoing hemodialysis to achieve and maintain molecular CR. ATO can be used safely with careful monitoring of electrolytes and electrocardiograms without measuring the serum ATO concentration. Further research involving a larger number of similar cases is required to verify the appropriate number of consolidation therapy sessions.

## CONFLICT OF INTEREST

We declare no conflict of interest.

## AUTHOR CONTRIBUTIONS

AH: designed this project and wrote the manuscript. AH, YT, and IS: managed the patient. YT: supervised this project and critically revised the manuscript. All authors approved the final version of the manuscript.

## ETHICAL APPROVAL

We confirmed that the manuscript has been read and approved by all named the authors. The protection of intellectual property associated with the manuscript had been in our consideration.

## Data Availability

The authors declare that data supporting the findings of this study are available within the article.
